# Simulation and Synthesis of Cobalt (Co) Nanoparticles by Gamma Radiation Technique

**DOI:** 10.3390/mi14071383

**Published:** 2023-07-06

**Authors:** Elham Gharibshahi, Shahidan Radiman, Ahmadreza Ashraf, Elias Saion, Leila Gharibshahi, Sina Ashraf

**Affiliations:** 1 Department of Electrical and Computer Engineering, University of Texas at San Antonio (UTSA), One UTSA Circle, San Antonio, TX 78249, USA; 2 School of Applied Physics, Faculty of Science and Technology, National University of Malaysia (UKM), UKM Bangi, Selangor 43600, Malaysia; 3 Department of Physics, Faculty of Science, University of Putra Malaysia (UPM), UPM Serdang, Selangor 43400, Malaysia; 4 School of Mathematics, Science and Engineering, University of the Incarnate Word (UIW), 4301 Broadway, San Antonio, TX 78209, USA

**Keywords:** radiolytic synthesis, cobalt nanoparticles, optical properties, conduction energy, simulation using quantum mechanical calculations

## Abstract

Cobalt nanoparticles were synthesized using the gamma radiolytic technique, and the particle size was found to be reduced from 12±1 to 7±1 nm by increasing the dose from 10 to 60 kGy. The UV-visible absorption spectra were measured and exhibited a steady absorption maxima at 517 nm in the UV region, which blue-shifted toward a lower wavelength with a decrease in particle size. By taking the conduction electrons of an isolated particle that are not entirely free but are instead bound to their respective quantum levels, the optical absorption of the cobalt nanoparticles can be calculated and simulated via intra-band quantum excitation for particle sizes comparable to the measured ones. We found that the simulated absorption maxima of electronic excitations corresponded to the measured absorption maxima. Moreover, the structural characterizations were performed utilizing dynamic light scattering (DLS), transmission electron microscopy (TEM), and X-ray diffraction (XRD).

## 1. Introduction

Nanostructured materials possess different properties relative to their macroscopic-structured counterparts. These materials play a significant role in technology as they are incorporated into components, systems, and devices [1,2,3,4]. These properties may lead to new applications with increased functionalities. Metallic nanoparticles have many applications in electronics, catalysis, photonics, and biochemical sensing and imaging [5,6]. They have attracted significant attention in the field of catalysis due to their considerable catalytic activity [7,8]. Scientists have investigated colloidal metal nanoparticle catalysts in various reactions in homogeneous catalysis, including the decomposition of hydrogen peroxide [7,9] and hydrazine in aqueous solutions [9].

The properties of nanostructured magnetic materials differ from bulk materials because their small dimensions allow for quantum effects, as well as domain suppression and configurational anisotropy [10,11].

Among the various types of nanostructures, magnetic nanomaterials based on alloys of metals of the iron subgroup (Fe and Co) and their various oxide forms have unique magnetic, electronic, and optical properties [12,13]. For instance, cobalt ferrite (CoFe_2_O_4_) magnetic nanomaterials have been studied, including the investigation of the magnetic and electrical properties of CoFe_2_O_4_ spinel ferrite materials [14]. Additionally, other Co-based compound research includes the study of the efficiency of thermally annealing nanostructures for phase transformations of the FeCo–Fe_2_CoO_4_/Co_3_O_4_ spinel type [12].

Magnetic particles of nanometer size have received considerable attention for progressing fundamental and technological interests due to their potential applications in motors, electrical power transformation, magnetic fluids, and magnetic resonance imaging [15,16,17,18,19].

Among magnetic materials, cobalt nanoparticles have received considerable attention due to their high saturation magnetization and high coercivity [5,20]. Moreover, cobalt nanoparticles display structure-dependent magnetic and electronic properties.

There have been numerous approaches to synthesizing metal nanoparticles such as cobalt nanoparticles, including thermal decomposition [21,22], gas vapor condensation [23,24], solution phase metal salt reduction [25,26], the water–oil inverse micelle technique [27], solvothermal synthesis [28], solution phase decomposition [21,29,30], the double agent reduction method [31], the electrodeposition method [32,33,34,35], the casting method [36], the X-ray method [37], and gamma irradiation [5,38,39,40,41,42]. Among these techniques, the gamma irradiation method presents some advantages over conventional methods. It is an uncomplicated process that provides fully reduced and very pure metal nanoparticles which are free from other products or reducing agents [42,43,44,45,46].

The radiation method has significantly contributed to progress in various types of research [6,47,48,49,50,51]. The interaction of ionizing radiation, such as gamma rays, with aqueous solutions leads to the generation of randomly distributed reducing and oxidizing agents with large redox potentials. Through the use of scavengers, the environment in the solution can be modified to generate a high concentration of reducing agents. These species can readily react with solvated metal ions and decrease their oxidation state [52,53,54]. Subsequently, the metallic atoms obtained in the solution nucleate into clusters and grow into particles [6,51,52,53,54].

The optical properties of metal nanoparticles have been of interest in physical chemistry [55]. Currently, the optical absorption of metal nanoparticles is subordinated through their localized surface plasmon resonances (LSPRs), which are related to the collective coherent oscillation of conduction electrons in resonance accompanied by the incident electromagnetic wave and are regulated via the dielectric constants of the particles and the medium [56,57]. In addition, the explanation for the catalytic action of metal nanoparticles is still unknown [58] since the oscillating conduction electrons of metal nanoparticles may not be able to participate in a photocatalytic process. Hence, a new theory of metal nanoparticles is necessary to explain the photocatalytic action.

This article presents a procedure to synthesize colloidal cobalt nanoparticles which are acquired using a gamma radiolytic technique. The optical absorption of the prepared nanoparticles was measured and compared with the simulated values. In addition, the effect of dose on the particle size of the Co nanoparticles was investigated. Furthermore, TEM, DLS, and UV-Vis spectroscopy were employed to characterize the structural and optical properties of the cobalt nanoparticles.

## 2. Experimental

### 2.1. Materials

Cobalt dichloride hexahydrate (CoCl_2_·6H_2_O) was used as a metal precursor, poly(vinyl pyrrolidone) (PVP) was used as a capping agent to diminish the agglomeration of Co nanoparticles, isopropyl alcohol (IPA) was used as a radical scavenger of hydrogen and hydroxyl radicals, and tetrahydrofuran (THF) and deionized water were used as solvents for the metal complex and polymer, respectively. Nitrogen gas in the highly pure form of 99.5% was used to remove oxygen from the solution.

### 2.2. Procedure

First, 22.44 $\times 10^{-4}$ M of CoCl_2_·6H_2_O was dissolved in 50 mL of THF before being introduced into an aqueous solution of 3 g of PVP in 150 mL of deionized water. After adding 25 mL of IPA, the solution was magnetically stirred and bubbled with 99.5% nitrogen gas for 1 h before filling several glass tubes with the mixed solution. The specimens were irradiated with ^60^Co gamma rays at various doses of 10, 20, 30, 40, 50, and 60 kGy.

### 2.3. Characterization

The optical absorbance spectrum was measured using a UV–visible spectrophotometer (Perkin Elmer, Lambda 900 UV/Vis, Waltham, MA, USA). There was no treatment for the irradiated specimens prior to the optical absorption analysis. The particle size and the size distribution were determined using a HITACHI transmission electron microscope (TEM; H 7500), employing an accelerating voltage of 100 kV. The TEM specimens were processed by positioning a drop of the irradiated solution on a copper grid and allowing the sample to dry naturally overnight.

Moreover, the particle size and the size distribution of the specimens were measured utilizing a Zetasizer Nano ZS instrument (Malvern Instruments, Malvern, UK) which was equipped with a 4 mW He-Ne laser [59]. The intensity-averaged particle diameters and the polydispersity index (PDI) values (an estimate of the distribution width) were calculated from the cumulate analysis, as defined in the ISO 13321 (International Organization for Standardization) [60].

The intensity size distributions were acquired from an analysis of the correlation functions using the general purpose algorithm in the instrument software. This algorithm is founded upon a non-negative least squares fit [61,62].

## 3. Results and Discussion

### 3.1. Experimental Process

#### Radiolytic Reduction Method

The interaction of gamma photons with matter includes several distinctive procedures depending on the energy of the photons and on the atomic number of the medium and its density. In an aqueous solution, 1.25 MeV ^60^Co gamma rays interact with matter, resulting in the formation of secondary electrons. 

These free and energetic electrons can induce several reactive species, such as a large number of the hydrated electrons ($e_{aq}^{-}$), hydroxyl radicals (OH^•^), and hydrogen radicals (H^•^) that are produced during the radiolysis of aqueous solutions via irradiation (Equation (1)).

The hydrated electrons ($e_{aq}^{-}$) possess reductive properties as they have a very negative reduction potential of E_o_ = −2.87 V [52,54], while the OH^•^ possess the strongest oxidative properties with an oxidation potential of E_o_ = +2.73 V [53,54]. The hydrogen radicals (H^•^) are a strong reducing agent with a redox potential of E_0_ = −2.3 V [52].

(1) $$H_{2}O\;\to \;e_{aq}^{-},\;H_{3}O^{+},\;H^{•},\;{OH}^{•},\;H_{2},H_{2}O_{2}\;\;\;\;\text{(radiolysis of water by gamma rays)}$$

*Nucleation and Growth*

The hydrated electrons arising from the radiolysis of water can easily reduce all metal ions to zerovalent atoms (M^0^). The atoms, which are formed via the radiolytic method, are distributed homogeneously throughout the solution. This is a result of the reducing agents generated by the radiation, which can deeply penetrate the sample and randomly reduce the metal ions in the solution. These newly formed atoms act as individual centers of nucleation and further coalescence. The binding energy between two metal atoms or atoms with unreduced ions is stronger than the atom–solvent or atom–ligand bond energies [51]. Therefore, the atoms dimerize when encountering or being associated with the excess metal ions [53]:

(2) $$M^{0}+M^{0}\to M_{2}$$

(3) $$M^{0}+M^{+}\to M_{2}^{+}$$

The charged dimer clusters $M_{2}^{+}$ may further be reduced to form a center of cluster nucleation. The competition between the reduction of free metal ions and the absorbed ones could be controlled by the rate of reducing agent formation [63]. The reduction of ions that are fixed on the clusters favors cluster growth rather than the formation of newly isolated atoms. The bonding between clusters with unreduced ions or two charged clusters is also strong, and these association processes are fast [53].

*Formation of Co Nanoparticles*

In the formation of cobalt nanoparticles, the electrons liberated via gamma irradiation, in turn, reduce the metal ions of ${Co}^{2+}$ into zerovalent metals of Co^0^, which is known as the nucleation process. The formation of Co nanoparticles can be described by the following reactions [5,52]:

(4) $$Co{Cl}_{2}⟶{Co}^{2+}+2{Cl}^{-}\;\;\;\;\text{(ion dissociation)}$$

(5) $${Co}^{2+}+2e_{aq}^{-}⟶{Co}^{0}\;\;\;\;\text{(first nucleation)}$$

(6) $${Co}^{0}+{Co}^{0}⟶{Co}_{2}^{0}\;\;\;\;\text{(agglomeration)}$$

(7) $${Co}_{m}^{0}+{Co}^{0}⟶{Co}_{m+1}^{0}\;\;\;\;\text{(agglomeration)}$$

In the synthesis procedure, cobalt dichloride dissociates into positive ${Co}^{2+}$ cations and negative $2{Cl}^{-}$ anions, Equation (4). The hydrated electrons reduce ${Co}^{2+}$ into zerovalent Co atoms (${Co}^{0}$) via the nucleation process, Equation (5). A number of ${Co}^{0}$ atoms can agglomerate to form ${Co}_{2}^{0}$ or ${Co}_{m+1}^{0}$ nanoparticles, as shown in Equations (6) and (7), respectively.

The hydroxyl radicals formed in the radiolysis of water (Equation (1)) are able to oxidize the ions or the atoms into a higher state of oxidation. To prevent this oxidation, the radicals were scavenged efficiently by adding isopropanol.

The radicals of hydroxyl and hydrogen are strong reducing agents in an aqueous colloidal solution; hence, the addition of isopropanol to the precursor solutions was required.

IPA scavenged radicals of hydroxyl and hydrogen and simultaneously was changed into IPA radicals, Equations (8) and (9), which eventually reduce ${Co}^{2+}$ ions into ${Co}^{0},$ as shown in Equation (10).

(8) $${OH}^{•}+{CH}_{3}-CH\left(OH\right)-{CH}_{3}⟶{CH}_{3}-C^{•}\left(OH\right)-CH_{3}+H_{2}O\;\;\;\;\text{(radicals formation)}$$

(9) $$H^{•}+CH_{3}-CH\left(OH\right)-{CH}_{3}⟶CH_{3}-C^{•}\left(OH\right)-CH_{3}+H_{2}\;\;\;\;\text{(radicals formation)}$$

(10) $${Co}^{2+}+2\left[CH_{3}-C^{•}\left(OH\right)-CH_{3}\right]⟶2\left[CH_{3}-CO-CH_{3}\right]+{Co}^{0}+2H^{+}\;\;\;\;\text{(nucleation)}$$

For metal and alloy composite samples, the appearance of oxygen can lead to great changes in the electronic parameters that will seriously affect the practical application of the materials obtained. It is well known that complex transition metal compounds easily oxidize [64,65].

In this research, there was no oxygen in the prepared samples since the final solutions were already bubbled with 99.5% nitrogen gas to remove oxygen [42,66,67]. Moreover, the hydroxyl radicals that can oxidize the ions were unable to oxidize them because the radicals were scavenged by the addition of isopropanol [66,67].

### 3.2. DLS Results

**Figure 1:**
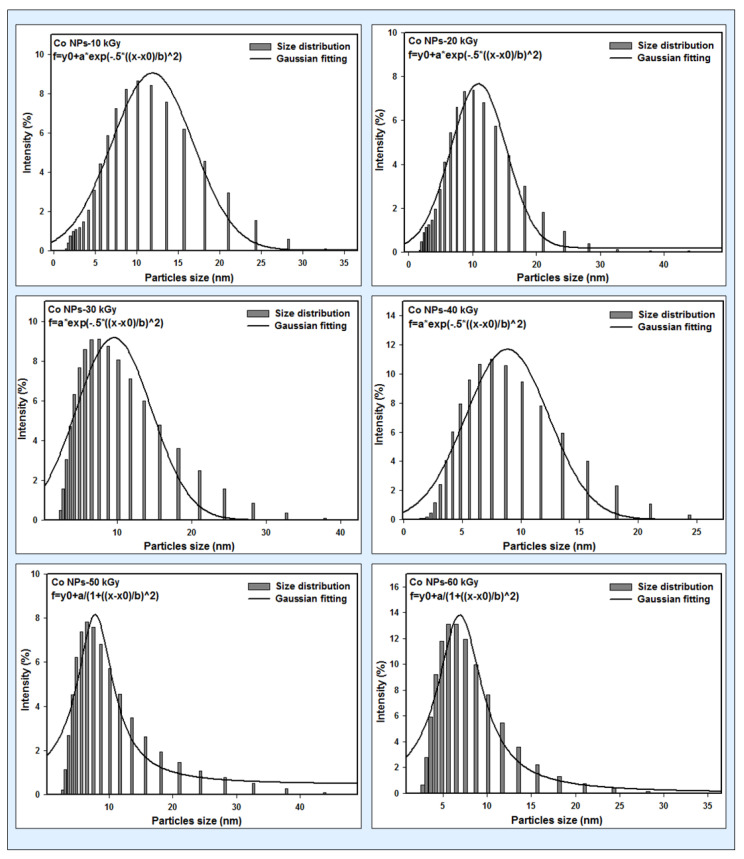
The size distribution of Co nanoparticles at different radiation doses was measured using the DLS technique.

The size distribution of the Co nanoparticles at different radiation doses was determined using a dynamic light scattering spectrophotometer. Dynamic light scattering was carried out to monitor the hydrodynamic sizes of the magnetic nanoparticles. The analyses based on the DLS particle size analyzer and the results show that the average size of the Co nanoparticles at different doses was approximately between 7±1 nm and 12±1 nm, as demonstrated in Figure 1.

### 3.3. TEM Images of Colloidal Co Nanoparticles

**Figure 2:**
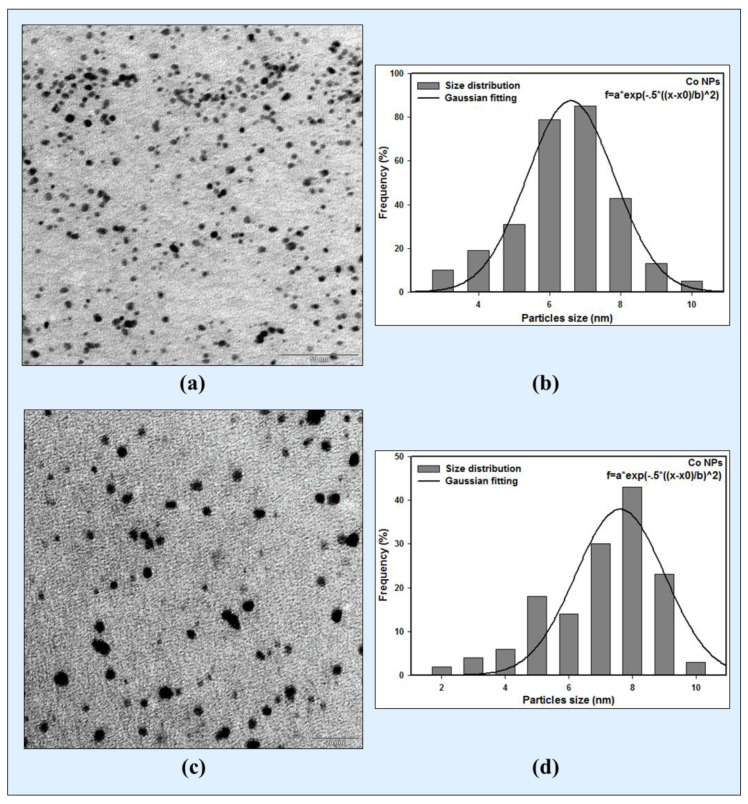
(**a**) TEM micrograph and (**b**) size distribution and Gaussian fitting of Co nanoparticles, irradiated with 60 kGy. (**c**) TEM micrograph and (**d**) size distribution and Gaussian fitting of Co nanoparticles irradiated with 50 kGy.

The TEM images of the particle distribution of the Co nanoparticles synthesized via the gamma radiolytic reduction methods are exhibited in Figure 2a,c for doses of 60 and 50 kGy, respectively.

The TEM images demonstrate well-dispersed spherical particles. The average particle sizes of the Co nanoparticles synthesized at 60 and 50 kGy, as determined from the Gaussian fitting of the size histogram, shown in Figure 2b,d, are 6.6±0.5 nm and 7.6±0.5 nm, respectively.

The comparison between the results of TEM and DLS revealed that the sizes of the Co nanoparticles synthesized at 50 kGy and 60 kGy using DLS are 7.8 nm and 6.9 nm, which are approximately close to the TEM results. It was discovered that the measurements made via DLS utilizing intensity distribution presented good results when compared to the TEM results.

### 3.4. XRD Analysis of Co Nanoparticles

**Figure 3:**
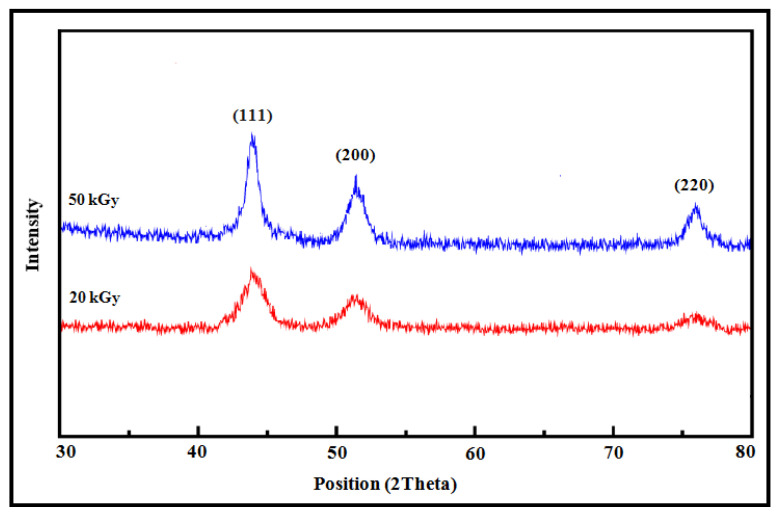
XRD patterns of Co nanoparticles synthesized via the gamma radiolytic method at doses of 20 kGy and 50 kGy.

Figure 3 indicates the XRD patterns of the Co nanoparticles synthesized via the gamma radiolytic reduction method at doses of 20 kGy and 50 kGy. The XRD peaks were matched perfectly with the (111), (200), and (220) crystalline planes of the face-centered cubic (FCC) structure of cobalt nanoparticles. The intensity of these peaks increased with an increasing dose, which is probably due to the increase in the number of Co nanoparticles. 

The mean crystallite size of Co nanoparticles may be assessed from the width at the XRD peak utilizing Scherrer’s equation [68], which is provided by:

(11) $$D=\frac{k\lambda }{\beta Cos\theta }$$

where *D* stands the average crystallite size, *k* is the particle shape factor that alters with the method of taking the width and shape of the crystallite (*k* = 0.89), *λ* is the X-ray wavelength used (0.1542 nm), *β* is the angular line width of the half-maximum intensity, and *θ* is Bragg’s angle in degrees.

The average crystallite sizes of the Co nanoparticles were calculated using the dominant (111) reflection of the XRD pattern and were found to be 11±1 nm and 8±1 nm for Co nanoparticles synthesized at 20 kGy and 50 kGy, respectively. The particle sizes are in good agreement with the values determined by the TEM and DLS methods.

### 3.5. Optical Properties

#### 3.5.1. Experimental

**Figure 4:**
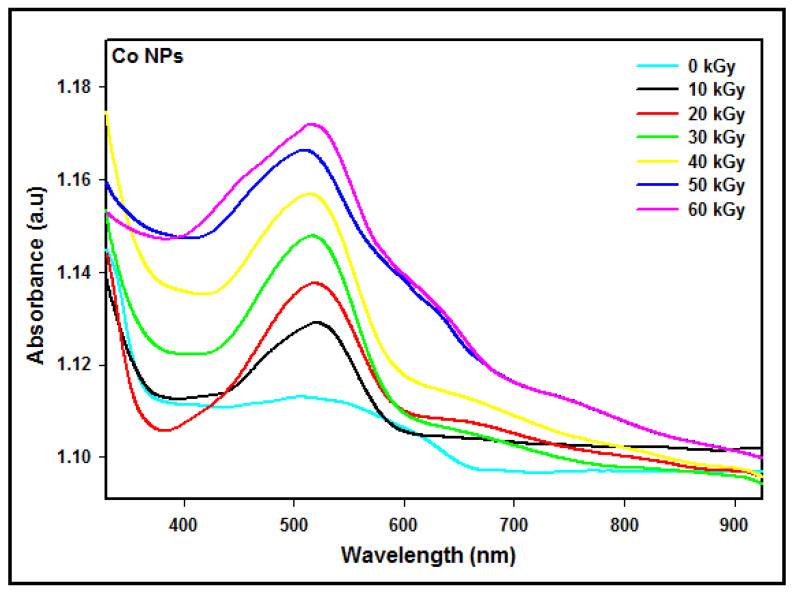
UV–visible absorption spectra of colloidal Co nanoparticles synthesized at doses from 10 to 60 kGy.

The absorption spectra of the Co nanoparticles synthesized at different radiation doses from 10 kGy to 60 kGy are shown in Figure 4. The results showed that the absorbance was enhanced via increasing the dose owing to the number of Co nanoparticles multiplied with the increasing dose. Since the number of ${Co}^{2+}$ ions that were reduced to zerovalent ${Co}^{0}$ atoms increased by increasing the radiation dose, this shows that the number of Co nanoparticles of smaller sizes increased with an increasing dose.

In addition, the absorption maximum was blue-shifted toward a lower wavelength with an increasing dose, demonstrating that the particle size decreases as the dose increases. By decreasing the particle size, the conduction electrons are less attracted to the core of the particle, and after receiving photon energy, they can be excited to higher energy levels, yielding the blue shift.

The conduction band energy *E* of metal nanoparticles may be acquired from the absorption maxima $\lambda_{max}$ according to ${E=hc/\lambda }_{max}$, where *h* is Planck’s constant and *c* represents the speed of light. The conduction band energy represents the amount of energy required to excite the conduction electrons from the lowest energy state to higher energy states impacted by UV-visible electromagnetic radiation.

The maximum absorption peaks and conduction energies of the Co nanoparticles synthesized at doses from 10 to 60 kGy are exhibited in Table 1.

#### 3.5.2. Theoretical Model and Simulation

The theory of the light absorption of metal nanoparticles was presented using classical electrodynamics, which delineated the coherent oscillation of conduction electrons known as the localized surface Plasmon resonance (LSPR) [69]. However, the physical parameters of the particles are not expressed in the classical formulation.

The current model is an effort to elucidate the UV experimental results of the Co nanoparticles. Our approach is to observe specific Co nanoparticles at various sizes acquired from the experiment. The common factor of the optical properties of the Co nanoparticles would be the number of conduction electrons possessed by an individual Co particle which is directly contributing to the absorption spectrum.

A theory of metal nanoparticles should consider the particle’s geometric structure and the electronic structures of metallic atoms. The conduction electrons of metal nanoparticles rely on several physical parameters, including particle size, crystalline structure, crystalline constant, the number of conduction electrons, and their quantum numbers.

The optical absorption of metal nanoparticles may be depicted via quantum mechanical interpretation through intra-band excitations of conduction electrons.

The density functional theory of conduction electrons may be achieved from the Thomas–Fermi–Dirac–Weizsacker model, which is fundamental for all ground-state properties, such as the absorption of metal nanoparticles. The Euler–Lagrangian equation *E*[*ρ*(*r*)] of this model can be written as:

(12) $$\frac{5}{3}C_{k}\int {\rho \left(r\right)}^{2/3}dr+\frac{\eta }{8}\left[\frac{{\left|\nabla \rho \left(r\right)\right|}^{2}}{\rho^{2}\left(r\right)}-2\frac{\nabla^{2}\rho \left(r\right)}{\rho \left(r\right)}\right]+v\left(r\right)+\int \frac{\rho \left(\overset{´}{r}\right)}{\left|r-\overset{´}{r}\right|}d\overset{´}{r}-\frac{4}{3}C_{e}\int {\rho \left(r\right)}^{1/3}dr=E_{0}$$

where *ρ*(*r*) is the density of conduction electrons of a Co nanoparticle, *E*_0_ is the Fermi energy, and *r* is the displacement of conduction electrons from the center of the spherical nanoparticle, which is dependent on the Bohr radius *a*_0_, the atomic number *Z*, and the principle, angular, and spin quantum numbers *n*, *l*, and, *s*, respectively.

In Equation (12), the first term is the Thomas–Fermi kinetic energy of the homogeneous free electron gas, with *C_k_* being a constant. The second term is the Weizsacker correction to the Thomas–Fermi kinetic energy via the inclusion of the exchange and correlation energy terms of inhomogeneous electron density, with η as a constant. The third term is the potential energy of the system. The fourth term represents the classical Coulomb potential energy of electron–electron interactions. The last term is the non-classical exchange–correlation energy, involving all the remaining quantum effects not captured by the kinetic energy and the classical Coulomb potential, and *C_e_* is the Thomas–Fermi–Dirac non-classical exchange–correlation energy constant. The association between the density *ρ*(*r*) and absorption σ(*r*) may be written as *ρ*(*r*) ≈ (*Z*/*σ*(*r*))^3/2^, where *Z* is the atomic number. The transformation of the density energy functional *E*[*ρ*(*r*)] into absorption energy functional *E*[*σ*(*r*)] can be attained mathematically.

In the calculation and simulation, each conduction electron is allowed to follow the excitation event interactively from the lowest energy state to higher energy states near the Fermi level to create the absorption spectrum.

**Figure 5:**
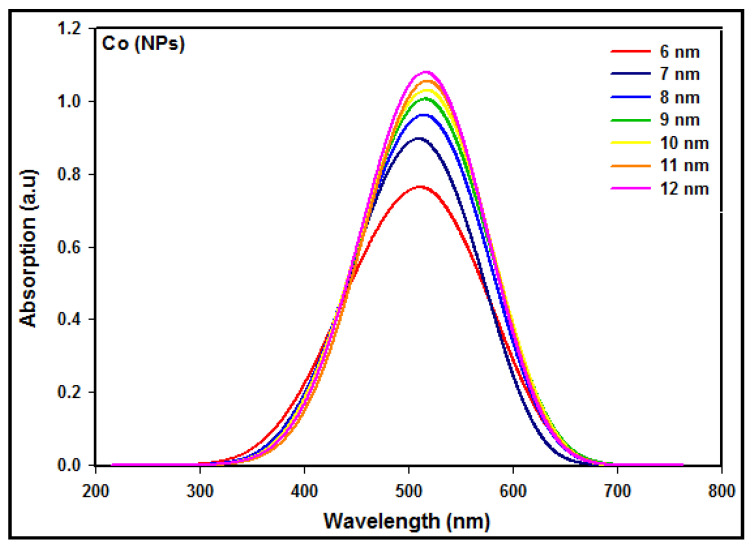
Calculated and simulated absorption spectra for isolated Co nanoparticles of different diameters.

Figure 5 displays the calculated and simulated absorption spectra, showing the absorption maxima designed for an isolated Co nanoparticle of a diameter from 7±1 to 12±1 nm.

The absorption peak is accredited to the intra-band quantum transitions from the lowest energy state of $\left\{n=3;\;l=2\right\}$ to the higher energy states of $\left\{n\geq 4;\;∆l=0,\;\pm 1;\;∆s=0\right\}$. It is clear that the theoretical absorption spectra (Figure 5) and the experimental absorption spectra (Figure 4) are not similar in terms of the maximum intensity and the peak width. The reason is that the calculated and simulated spectra were based on an isolated single Co nanoparticle of a given diameter, while the measured spectra were obtained from many synthesized Co nanoparticles of different diameters.

The absorption spectrum calculated and simulated through quantum mechanical treatment demonstrates that the absorption maximum is red-shifted from 509.63 to 517.43 nm, with an increase in the particle size from 6 to 12 nm. An increase in the absorption maxima displays a quantum confinement effect of nanomaterials. The calculated conduction energies and maximum absorption peaks of the Co nanoparticles are shown in Table 2.

### 3.6. Interpretation between the Measured and Simulated Absorption Spectra

According to Figure 4, the UV experimental absorption spectra reveal that the absorption increases with the number of particles present in the medium. The absorption maximum is blue-shifted toward a lower energy wavelength as the particle size diminishes at higher doses. On the other hand, in Figure 5, the calculated and simulated absorption spectra include all conduction electrons present in the particle. Hence, a larger particle size with more conduction electrons would yield a higher intensity absorption spectrum since the number of iterations needed enhances larger particle sizes. The absorption maximum is red-shifted as the particle size enhances. The most important knowledge of the experimental and theoretical absorption spectra is that the maximum absorption peaks for a given particle size are coincidently in good agreement between the measured and simulated results indicated in Table 3.

**Figure 6:**
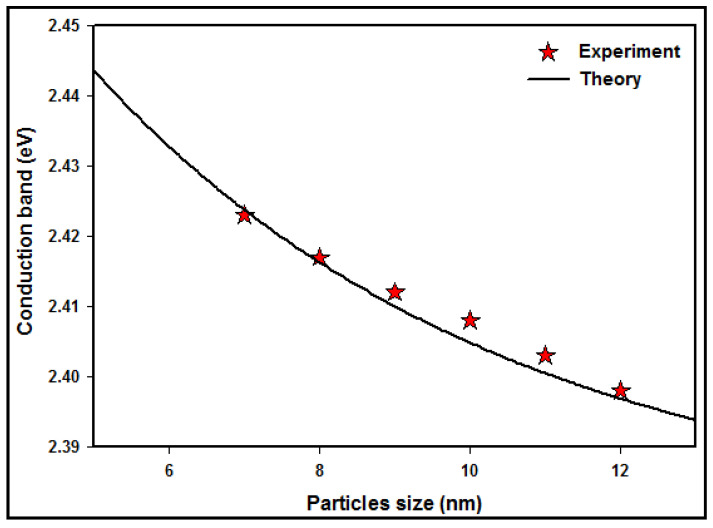
Comparison between the experimental and theoretical absorption maxima of Co nanoparticles at different particle sizes.

A comparison between the experimental and theoretical absorption maxima of Co nanoparticles at various particle sizes is demonstrated in Figure 6. The conduction band data decreases with increasing particle size due to the quantum confinement effect of the conduction electrons of the Co nanoparticles.

The conduction band of the experiment reduced from 2.423 to 2.398 eV through the increase in nanoparticle size from 7±1 nm to 12±1 nm. Furthermore, the theoretical conduction band decreased from 2.433 to 2.396 eV via the increasing particle size.

The number of atoms is numerous for larger particles, and so the conduction electrons are more attracted to the protons of the particle and decrease the conduction band energy, whereas for the smaller particle sizes, there are fewer atoms, and the electrons are less attracted to the core, increasing the conduction band energy of the Co nanoparticles.

## 4. Conclusions

The Co nanoparticles exhibited absorption maxima in the region of 517 nm, which is blue-shifted with a decrease in particle size. The agreement between the measured and theoretical absorption peaks indicates that those measured absorption peaks come from the intra-band excitation of conduction electrons of {*n* = 3; *l* = 2} quantum energy states to higher quantum energy states. The reason must be associated with the dose, which produces a particular particle size, which is the most important parameter in the calculation to compare with the experimental results.

Hence, we conclude that in metal nanoparticles, the intra-band quantized excitation of conduction electrons could occur from the lowest energy states to the highest energy states when the particles receive energy from electromagnetic radiation. The new theory is fundamentally reliable for the quantum mechanical description of the absorption phenomenon of metal nanoparticles.

## Figures and Tables

**Table 1:** Average particle sizes, maximum absorption peaks, and conduction energies of Co nanoparticles synthesized via the radiolytic reduction method at doses of 10 to 60 kGy.

Dose (kGy)	Particle Size (nm)	Absorption Peak *λ*_max_ (nm)	Conduction Energy (eV)
10	11.9±0.8	517	2.398
20	10.9±0.6	516	2.403
30	9.5±1.4	515	2.408
40	8.8±1.4	514	2.412
50	7.8±1.1	513	2.417
60	6.9±1.7	512	2.423

**Table 2:** The theoretically calculated conduction energies, maximum absorption peaks, and average particle sizes of Co nanoparticles.

Particle Size (nm)	Absorption Peak *λ*_max_ (nm)	Conduction Energy (eV)
6	509.63	2.433
7	511.52	2.424
8	513.52	2.415
9	514.47	2.410
10	515.41	2.406
11	516.42	2.401
12	517.43	2.396

**Table 3:** The simulated and measured values of the absorption peaks.

Particle Size (nm)	Absorption Peak *λ*_max_ (nm)		
	Experiment	Theory	Difference (%)
12	517	517.43	0.083
11	516	516.42	0.081
10	515	515.41	0.080
9	514	514.47	0.091
8	513	513.52	0.101
7	512	511.52	0.094

## Data Availability

The data presented in this study are available on request from the corresponding author.
